# Navigating preemptive and therapeutic donor lymphocyte infusions in advanced myeloid malignancies by high-sensitivity chimerism analysis

**DOI:** 10.3389/fonc.2022.867356

**Published:** 2022-08-17

**Authors:** Michael Stadler, Letizia Venturini, Ivonne Bünting, Elke Dammann, Eva M. Weissinger, Adrian Schwarzer, Christian Schultze-Florey, Steve Ehrlich, Dominik Markel, Catherina Lueck, Alexandra Gladysz, Tabea Fröhlich, Nouraldin Damrah, Gernot Beutel, Matthias Eder, Arnold Ganser, Lothar Hambach

**Affiliations:** Department of Hematology, Hemostasis, Oncology, and Stem Cell Transplantation, Hannover Medical School, Hannover, Germany

**Keywords:** Donor lymphocyte infusion, chimerism, allogeneic stem cell transplantation, alloreactivity, graft-versus-leukemia, graft-versus-host

## Abstract

Preemptive and therapeutic donor lymphocyte infusions (preDLI and tDLI) are widely used in relapsing and relapsed hematopoietic malignancies after allogeneic stem cell transplantation (alloSCT) to enhance the graft-versus-malignancy effect. However, in advanced myeloid malignancies, long-term survival after preDLI and tDLI remains low, reflecting our inability to master the double-edged sword of alloreactivity, balancing anti-neoplastic activity versus graft-versus-host disease (GvHD). We previously evaluated a quantitative PCR-based high-sensitivity chimerism (hs-chimerism) based on insertion/deletion polymorphisms instead of short tandem repeats, where increasing host chimerism in peripheral blood predicts relapse more than a month before clinical diagnosis, and declining host chimerism signals anti-host alloreactivity. Here we report 32 consecutive patients with advanced myeloid malignancies receiving preDLI or tDLI “navigated” by hs-chimerism (“navigated DLI”). We compared them to a historical cohort of 110 consecutive preDLI or tDLI recipients, prior to implementation of hs-chimerism at our institution (“controls”). Both groups were comparable regarding age, gender, conditioning, donor type, and time to DLI. With longer median follow-up of the navigated DLI group (8.5 versus 5 months), their landmark overall (64%) and disease-free survival (62%) at 2 years from first DLI compared favorably with controls (23% and 21%, respectively). Improved survival of navigated DLI was due to both reduced relapse incidence (38% versus 60%) and non-relapse mortality (17% versus 44%) at 2 years. Early relapse prediction by hs-chimerism allowed a preemptive approach in 28% of navigated DLI versus 7% in controls. Our results confirm hs-chimerism as a highly valuable tool for monitoring and steering immune interventions after alloSCT.

## Introduction

Alloreactivity induced by allogeneic stem cell transplantation (alloSCT) currently represents the strongest weapon against aggressive hematologic malignancies (1–3). However, a substantial number of patents still succumb to the underlying disease, despite many efforts pre and post-transplant to reduce relapse rate (4, 5). For patients with impending or overt relapse after alloSCT, augmenting alloreactivity is required, either by donor lymphocyte infusions (DLI) (6) or through second transplantation, apart from cytoreduction as needed. Unfortunately, both cell based approaches yield only limited success, with long term survival rates in advanced myeloid malignancies below 20% (7). Currently, we seem unable to precisely “dose” anti-host immune cells in order to control the deleterious effects of either too little or too much alloreactivity, i.e., subsequent relapse or severe graft-versus-host disease (GvHD) (8).

One prerequisite to steering alloreactivity is accurate, reliable, rapid, and affordable measurement of recipient/donor chimerism. For aggressive malignancies with high relapse dynamics, conventional chimerism techniques based on short tandem repeats (9) have been found not sensitive enough to detect impending relapse in useful time (10, 11). Next-generation sequencing approaches for minimal residual disease detection, on the other hand, are time-consuming, expensive and limited to previously identified disease markers which may even be lost during disease progression. We have recently evaluated and implemented a quantitative PCR-based high-sensitivity chimerism method (hs-chimerism) based on insertion/deletion polymorphism markers, which is applicable to more than 90% of recipient/donor pairs (12). With a sensitivity of 0.01%, this method allows prediction of relapse in whole blood samples more than a month in advance compared to conventional chimerism methods with sensitivities around 1%. Relapse is predicted by consistently increasing recipient signals over time, while declining recipient chimerism, e.g. when tapering immunosuppression or during GvHD, heralds anti-host alloreactivity. With a turn-over time of only one to two days and moderate cost, we are using this method for monitoring and guiding the post-transplant follow-up of our patients with aggressive malignancies.

Here, we report outcome data from our first 32 consecutive patients receiving preemptive DLI (preDLI, for impending relapse) or therapeutic DLI (tDLI, for established relapse) “navigated” by aid of hs-chimerism (navigated DLI), in comparison to a historical cohort of 110 consecutive recipients of preDLI or tDLI for advanced myeloid neoplasms, prior to implementation of hs-chimerism at our institution (controls).

## Patients and methods

### Design

This is a single center study of a prospective, non-randomized cohort with a historical control group.

### Patient cohorts

From 2016 to 2018, we validated and implemented a quantitative PCR-based, high-sensitivity chimerism method (hs-chimerism) using insertion/deletion polymorphism markers (12) instead of the short tandem repeat chimerism used previously at our institution. To evaluate the feasibility and efficacy of hs-chimerism in steering preDLI and tDLI, starting from 2019 we monitored all consecutive patients allotransplanted for advanced myeloid malignancies in whom the decisions on timing (initiating or delaying) of preDLI or tDLI were guided by hs-chimerism (“navigated DLI” group). All navigated DLI were performed from 2019 through 2021; data lock was December 31, 2021. For comparison, we choose all consecutive patients allotransplanted for advanced myeloid malignancies with preDLI and tDLI from 2001 to 2015 (“control group”). Patients’ characteristics are detailed in **Table 1**. Patients with preDLI and tDLI between 2016 and 2018 were excluded to avoid overlap with samples used for validation of hs-chimerism. (In addition to the navigated DLI group, 133 further patients with advanced myeloid malignancies were assessed by hs-chimerism between 2019 and 2021, but lacked significant increases in host chimerism and did not receive any DLI. Their characteristics are shown in the **Supplementary Table**). Data evaluation was in accordance with the declaration of Helsinki and amendments. All patients had given written informed consent to treatment, data analysis and publication prior to transplantation.

**Table 1:** Patients’ cohorts hs-chimerism navigated DLI historical control cohort.

Patients’ cohorts		hs-chimerism navigated DLI		historical control cohort	
N		32	*(100%)*	110	*(100%)*
Age at Tx	median	55 years		53 years	
	range	24 – 73 years		17 – 70 years	
Gender	female	13	(41%)	49	(45%)
	male	19	(59%)	61	(55%)
MDS, MPN, CML		5	(16%)	27	(25%)
AML, sAML, tAML		27	(84%)	83	(75%)
Genetic risk	standard	8	(25%)	41	(37%)
	high	24	(75%)	69	(63%)
Remission at Tx	CR1/CP1	18	(56%)	44	(40%)
	>CR1/CP1	14	(44%)	66	(60%)
Conditioning	MAC	12	(37%)	37	(34%)
	RIC	20	(63%)	73	(66%)
Donor	related	7	(22%)	24	(22%)
	unrelated	25	(78%)	86	(78%)
HLA match	10/10	28	(87%)	82	(75%)
	<10/10	4	(13%)	28	(25%)
Time Tx to 1st DLI	median	11 months		10 months	
	range	2 – 107 months		2–118 months	
DLI intention	preDLI	9	(28%)	9	(7%)
	tDLI	23	(72%)	101	(93%)
Follow-up after 1st DLI	median	8.5 months		5 months	
	range	1 – 33 months		0–211 months	

### hs-chimerism

Detailed procedures for hs-chimerism have previously been published (12, 13). Briefly, the method is based on human biallelic insertion/deletion polymorphism markers informative for recipient/donor genotype discrimination. Standard curves were created by subjecting serial dilutions of mixed DNA to real-time quantitative PCR. In the linear range, sensitivity was 0.01% (1 in 10.000). For individual hs-chimerism measurements, 300 ng of genomic DNA from patients’ whole blood were analyzed.

### Treatments

Treatment strategy was the same in both groups. Chimerism analyses were performed monthly during the first year after alloSCT and gradually reduced to once yearly after five years, except for situations of rising host chimerism as well as after DLI, when chimerism was measured more frequently. Chimerism assessment guided both adaptation of immunosuppression and DLI application. preDLI were performed for impending relapse, as detected by chimerism, cytogenetic or molecular diagnostics, but still with less than 5% bone marrow blasts. tDLI were carried out for overt relapse, mostly after cytoreductive treatment (chemotherapy, hypomethylating agents, targeted molecular therapies, as appropriate and available). Immunosuppression was off or stopped before DLI. Unstimulated donor peripheral blood leukaphereses were performed without *in vitro* manipulation. DLI were started at a dose of 1 x 10E7 CD3 positive donor cells per kg recipient’s body weight, and escalated in half-logarithmic steps by at least monthly intervals, unless precluded by symptoms or signs of incipient GvHD. In the navigated DLI group, declining host chimerism in serial measurements triggered delay or suspension of subsequent DLI in order to prevent unnecessary excess GvHD. First DLI doses were usually administered freshly after leukapheresis. Aliquots of donor lymphocytes were cryopreserved and thawed immediately prior to subsequent DLI. Premedication consisted of antihistamines, but no steroids. After DLI administration, patients were monitored by outpatient clinical visits every two weeks until further DLI, GvHD, disease remission, or progression.

### Statistics

Survival curves were calculated and drawn according to the method of Kaplan & Meier (14). Primary (overall survival) and secondary endpoints (disease-free survival, relapse incidence, non-relapse mortality, GvHD) were evaluated in landmark analyses from the day of first DLI. Due to the design of this study with a historical control cohort, and small numbers in the navigated DLI group, we refrained from calculations of significance and uni- or multivariate analyses, in favor of only descriptive and graphical comparisons between the cohorts.

## Results

To evaluate the feasibility of hs-chimerism in guiding DLI, we collected all 32 consecutive transplant recipients for advanced myeloid malignancies with preDLI or tDLI from 2019 until 2021 based on individual hs-chimerism results (“navigated DLI”). For comparison, all respective 110 consecutive patients with preDLI or tDLI from 2001 to 2015, prior to the implementation of hs-chimerism, served as a historical control cohort. (133 additional patients with advanced myeloid malignancies were monitored by hs-chimerism between 2019 and 2021, but without significant increases in host chimerism and without DLI.)

### Patients’ characteristics

Patients’ characteristics are detailed in **Table 1**. Both cohorts were well matched with respect to age, gender, conditioning intensity, donor type, and time from alloSCT to first DLI. Differences included more patients with aggressive diseases (AML/sAML/tAML; 84% versus 75%) and with high cytogenetic or molecular risk (75% versus 63%) for the navigated DLI group. On the other hand, fewer patients in the control cohort had reached complete remission (CR) before alloSCT (40% versus 56%) or had a fully matched donor (75% versus 87%).

(2019 – 2021 hs-chimerism patients without DLI were well matched with those of the navigated group, except for an even higher proportion of patients with adverse risk genetics: 85% versus 75%; see **Supplementary Table**).

### hs-chimerism patterns with DLI

**Figure 1:**
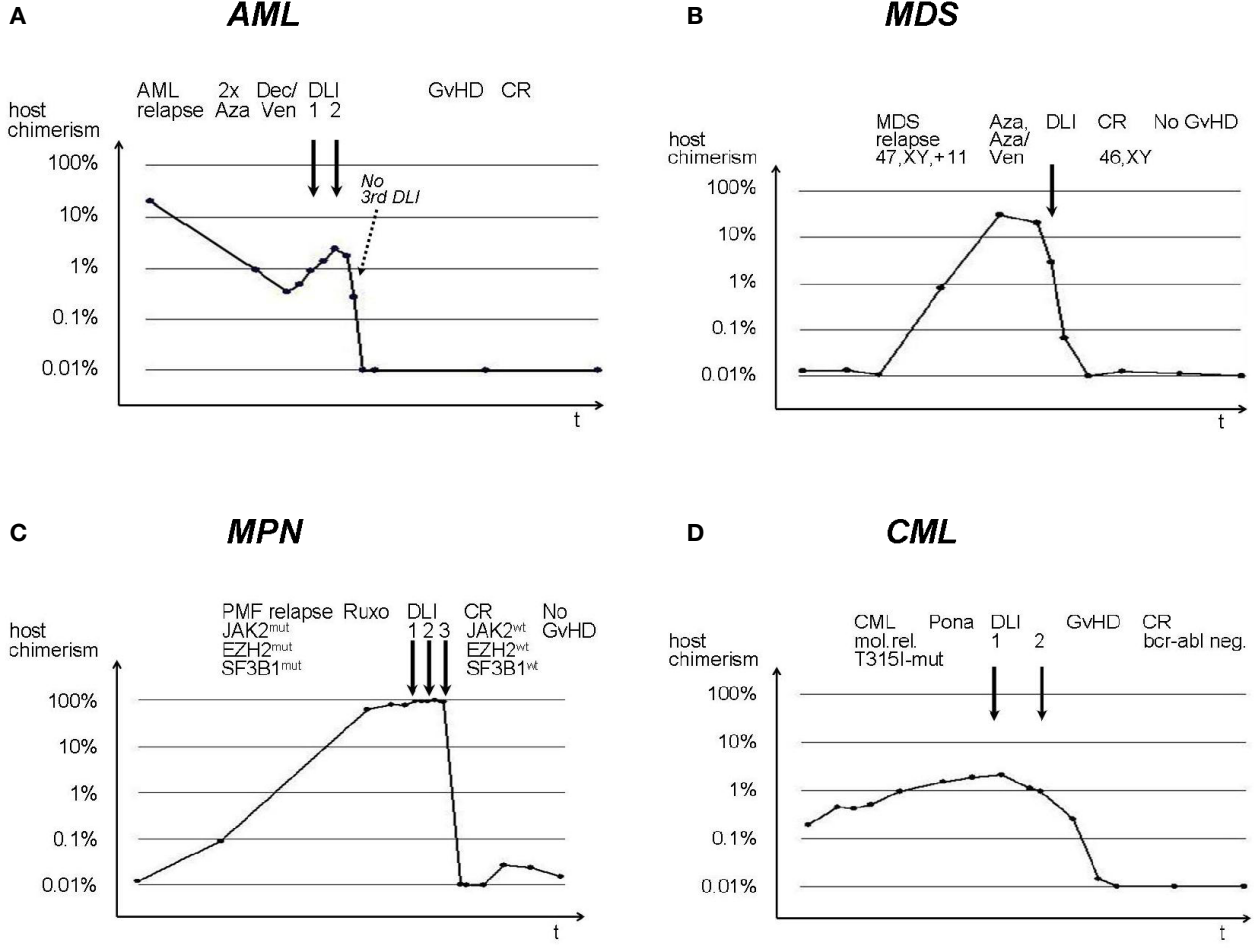
Individual hs-chimerism sequences in AML, MDS, MPN, and CML patients with navigated DLI. **(A)** AML, **(B)** MDS, **(C)** MPN, **(D)** CML. DLI are indicated by solid arrows. Aza, Azacytidine; Ven, Venetoclax; PMF, Primary Myelofibrosis; Ruxo, Ruxolitinib; Pona, Ponatinib; mut, mutation; wt, wild type. y-axes: host chimerism; x-axes: time after alloSCT.

Examples of individual hs-chimerism results from representative patients with navigated DLI for acute myeloid leukemia (AML), myelodysplastic syndrome (MDS), myeloproliferative neoplasia (MPN), and chronic myeloid leukemia (CML) are shown in **Figure 1**. In diseases with low relapse dynamics, the initial increase in host chimerism appeared protracted (**Figures 1B**–**D**). Decrease of host chimerism was rather slow with relapse pharmacotherapy (e.g. hypomethylating agents), but mostly precipitous after one or more DLI. Note in **Figure 1A** that decreasing host chimerism obviated application of subsequent DLI, even prior to clinical symptoms and signs of GvHD, thus sparing excess toxicity. DLI-induced anti-host alloreactivity cleared relapses at the cytogenetic and molecular levels (**Figures 1B**–**D**), and was not always associated with GvHD (**Figures 1B**, **C**).

### Follow-up

According to the study design, the range of follow-up after first DLI was much longer in the control cohort, starting as early as 2001 (211 months versus 33 months). However, the median follow-up after first DLI was longer in the navigated DLI group (8.5 months versus 5 months), already suggesting superior outcome.

### Outcome

**Figure 2:**
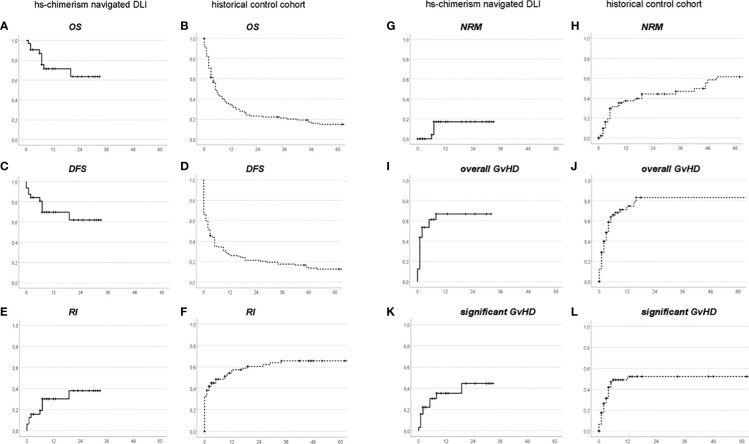
Landmark Overall Survival **(A, B)**, Disease-Free Survival **(C, D)**, Relapse Incidence **(E, F)**, Non-Relapse Mortality **(G, H)**, overall GvHD **(I, J)**, and significant GvHD **(K, L)**, from first DLI, in the hs-chimerism navigated group **(A, C, E, G, I, K)** or the historical control group **(B, D, F, H, J, L)**, respectively. x-axes: months after first DLI.

Landmark overall (OS) and disease-free survival (DFS) from first DLI are shown in **Figures 2A–D**. Whereas 2-year-OS and -DFS in the control cohort (23% and 21%, respectively) were well comparable with results from earlier studies (7), 2-year-OS and -DFS of the navigated DLI group reached 64% and 62%, respectively. This was due to improvements in both relapse incidence after first DLI (RI) with 2-year-RI for navigated DLI at 38% versus 60% for controls (**Figures 2E, F**) and non-relapse-mortality after DLI (NRM) with 2-year-NRM in the navigated DLI group at 17% versus 44% for the control cohort (**Figures 2G, H**). Accordingly, both overall GvHD (**Figures 2I**, **J**; navigated DLI 67% versus controls 83%) and significant GvHD (acute GvHD °III–°IV or chronic extensive GvHD; **Figures 2K**, **L**) at 2 years from first DLI favored the navigated DLI group (44% versus 52% in the control cohort).

(Outcomes at 2 years for the 2019 – 2021 hs-chimerism patients without DLI were: OS 91%, DFS 82%, RI 14%, NRM 5%, overall GvHD 75%, and significant GvHD 22%. Median follow-up was 14 (range, 2 to 33) months; see **Supplementary Figure 1**).

### preDLI and tDLI

**Figure 3:**
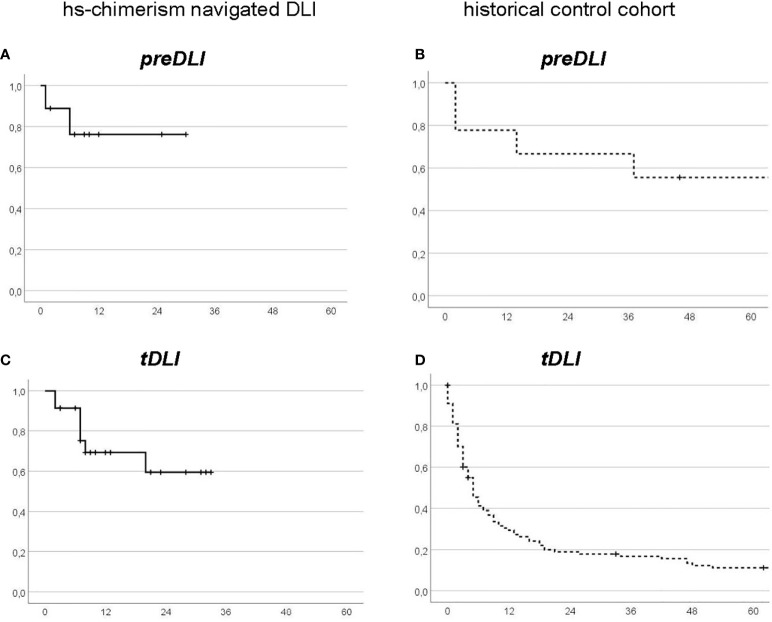
Landmark Overall Survival from first DLI, by DLI intention: preDLI **(A, B)** versus tDLI **(C, D)** in the hs-chimerism navigated group **(A, C)** or the historical control group **(B, D)**, respectively. x-axes: months after first DLI.

Outcome after preDLI appeared only moderately better in the navigated DLI group (2-year-OS 76% versus 67% in controls; **Figures 3A, B**), whereas in tDLI the difference was more pronounced (2-year-OS 60% in navigated DLI versus 19% in the control group; **Figures 3C, D**). This suggests that the main contribution to improvement by hs-chimerism occurred after established relapse. On the other hand, thanks to early relapse prediction, hs-chimerism enabled more often a preemptive approach (in 28% of navigated DLI versus 7% in controls) which by itself was associated with a better outcome. Thus, application of hs-chimerism resulted in a dual benefit “before and after” DLI.

## Discussion

Our data suggest that navigating preDLI and tDLI using hs-chimerism substantially improved outcomes, for both the individual patient and the whole cohort. Due to its high sensitivity, accuracy, reliability, rapid turnover, and moderate cost, this method enabled close monitoring of subsequent steps for relapse prevention and therapy timelier. This allowed us to modify therapeutic strategies prior to clinically relevant problems, e.g. to forgo additional DLI when host chimerism started to decline in order to reduce GvHD toxicity, or to schedule refractory patients for second alloSCT at an earlier time-point. For the navigated DLI group, steering of DLI by hs-chimerism paid off with consistently reduced relapse incidence, NRM, and GvHD, resulting in superior OS and DFS at 2 years from first DLI (almost tripled compared to our experience until 2015). The improvement through navigating by hs-chimerism was greatest in the tDLI setting, whereas preDLI patients had the benefit of early relapse prediction enabling a preemptive approach leading to already better outcome.

A clinical benefit of hs-chimerism monitoring was also obvious in the 133 patients with advanced myeloid malignancies not receiving DLI for lack of significant increases in host chimerism: with a median follow-up of 14 (range, 2 to 33) months, their outcome at 2 years was excellent with OS 91%, DFS 82%, RI 14%, and NRM 5% (**Supplementary Figure 1**), confirming our earlier observation of a very high negative predictive value (98-99% relapse exclusion) of the hs-chimerism (12), even in a cohort with substantial genetic risk (**Supplementary Table**). Interestingly, only 22% experienced significant GvHD despite an overall GvHD rate of 75%, suggesting that most of these patients had received just the right amount of mild GvL to prevent relapse.

Earlier studies on guiding preDLI by chimerism were hampered by limited sensitivity of conventional techniques and had focused on relapse prediction. Still, in pediatric leukemia patients, survival rates around 50% to 80% were reported for selected patients offered preDLI for increasing mixed chimerism (15, 16). In adults, preDLI for mixed chimerism detected by conventional or lineage specific chimerism analyses with sensitivities in the range of 1% or 0.1%, respectively, did not result in improved survival (17). However, a recent, large registry-based study on 192 patients receiving preDLI for minimal residual disease or mixed chimerism reported 51% and 63% OS, respectively, with no relapses beyond 3 years from first DLI (18).

Jacque et al. (11), using a high-sensitivity insertion/deletion chimerism quantitative real-time PCR technique, found lower relapse rates in patients offered immunomodulation for mixed chimerism (mostly tapering of immunosuppression), but lost both of their preDLI patients to acute GvHD °IV.

With regard to steering tDLI by chimerism analysis, we are unaware of any published studies.

Our analysis has several limitations:

First, a bias inherent to the design with a historical control cohort cannot be ruled out, although we undertook all efforts to minimize such bias by including all consecutive patients of the respective time periods into the cohorts.

Second, the size of the navigated DLI group is rather small, so individual outcomes might gain relatively more weight than in the larger control group (which, however, would apply for both favorable and unfavorable outcomes).

Third, the follow-up of the navigated group is still short. As can be seen from **Figure 2H**, there was a late increase beyond 3 years in NRM in the control cohort, probably as a consequence of chronic GvHD; a similar late increase cannot be excluded to occur in the future in the navigated DLI group as well (albeit less likely, due to the lower incidence of GvHD among navigated DLI patients). However, the median follow-up of the navigated DLI group already exceeded that of the control cohort at this time point, in line with superior outcome in the navigated DLI group.

Finally, with the control cohort originating in the remote past, there might be a bias through medical progress in favor of the more recent navigated group, e.g. based on more potent cytoreductive treatments before DLI. However, when splitting up the control cohort in half-decades (2001 to 2005: n = 42, 2006 to 2010: n = 31, 2011 to 2015: n = 37), 2-year-OS from first DLI remained at 28%, 17%, and 22%, respectively, with no progress over time to appreciate (**Supplementary Figure 2**). Furthermore, observing individual hs-chimerism data (**Figures 1A–D**), the effect of various therapies before DLI (azacytidine, decitabine, venetoclax, ruxolitinib, ponatinib) appeared rather small in comparison to the steep decline of host chimerism with alloreactivity. Venetoclax, the only drug not yet in use for myeloid relapse up to 2015, which therefore might have caused a bias between the navigated and control cohorts, did not change this pattern and was employed in less than half of the navigated group for cytoreduction prior to DLI. Thus, while a contribution of adjunctive medical therapies cannot be entirely ruled out, it would not be sufficient to explain the substantial outcome improvement observed with navigated DLI.

Taken together, hs-chimerism has proven a highly valuable tool for monitoring and steering immune interventions after alloSCT and deserves further evaluation and development.

## Data availability statement

The original contributions presented in the study are included in the article/**Supplementary Material**. Further inquiries can be directed to the corresponding author.

## Author contributions

Concept, design, and manuscript draft: MS. hs-chimerism: LV, IB, and LH. Patient care: MS, LH, AS, CS-F, SE, DM, CL, AGl, TF, ND, EMW, GB, ME, and AGa. Manuscript editing: LH, EMW, CS-F, CL, and ME. All authors contributed to the article and approved the submitted version.

## Acknowledgments

We thank Susanne Luther-Wolf for technical assistance with DNA preparation.

## Conflict of interest

The authors declare that the research was conducted in the absence of any commercial or financial relationships that could be construed as a potential conflict of interest.

## Publisher’s note

All claims expressed in this article are solely those of the authors and do not necessarily represent those of their affiliated organizations, or those of the publisher, the editors and the reviewers. Any product that may be evaluated in this article, or claim that may be made by its manufacturer, is not guaranteed or endorsed by the publisher.
