# Supplementary material for: Navigating preemptive and therapeutic donor lymphocyte infusions in advanced myeloid malignancies by high-sensitivity chimerism analysis
Source: Front Oncol. 2022 Aug 17;12:867356. doi: 10.3389/fonc.2022.867356 (PMC9428843; doi:10.3389/fonc.2022.867356)
Supplement: Supplementary file 1 [file DataSheet_1.docx]

**Supplementary Material**

**Suppl. Table**

Patients’ cohorts hs-chimerism navigated DLI hs-chimerism without DLI

2019 – 2021 2019 – 2021

N 32 *(100%)* 133 *(100%)*

Age at Tx median 55 years 58 years

range 24 – 73 years 21 – 76 years

Gender female 13 *(41%)* 59 *(44%)*

male 19 *(59%)* 74 *(56%)*

MDS, MPN, CML 5 *(16%)* 28 *(21%)*

AML, sAML, tAML 27 *(84%)* 105 *(79%)*

Genetic risk standard 8 *(25%)* 20 *(15%)*

high 24 *(75%)* 113 *(85%)*

Remission CR1/CP1 18 *(56%)* 67 *(50%)*

at Tx >CR1/CP1 14 *(44%)* 66 *(50%)*

Conditioning MAC 12 *(37%)* 38 *(29%)*

RIC 20 *(63%)* 95 *(71%)*

Donor related 7 *(22%)* 22 *(17%)*

unrelated 25 *(78%)* 111 *(83%)*

HLA match 10/10 28 *(87%)* 113 *(85%)*

<10/10 4 *(13%)* 20 *(15%)*

______________________________________________________________________

**Suppl. Figure 1**

OS, DFS, RI, NRM, overall and significant GvHD in hs-chimerism patients without DLI

______________________________________________________________________

A ***OS*** B ***DFS***


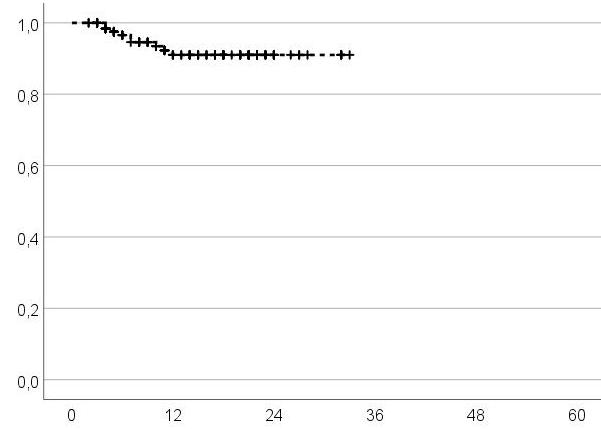

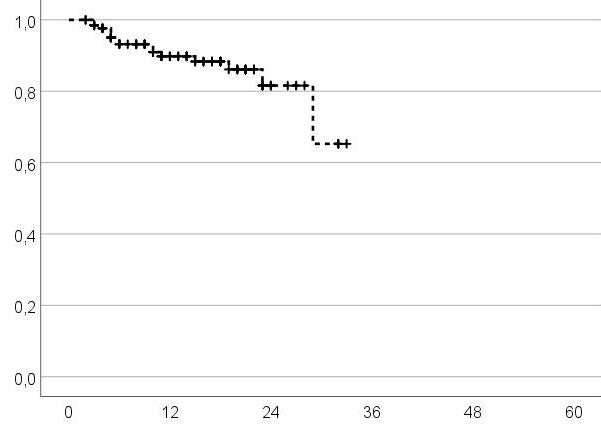


C ***RI*** D ***NRM***


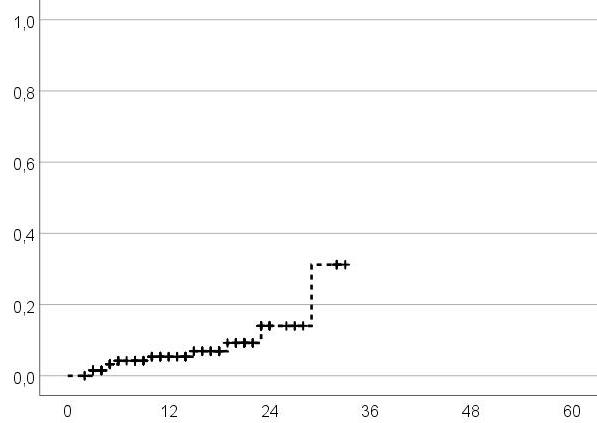

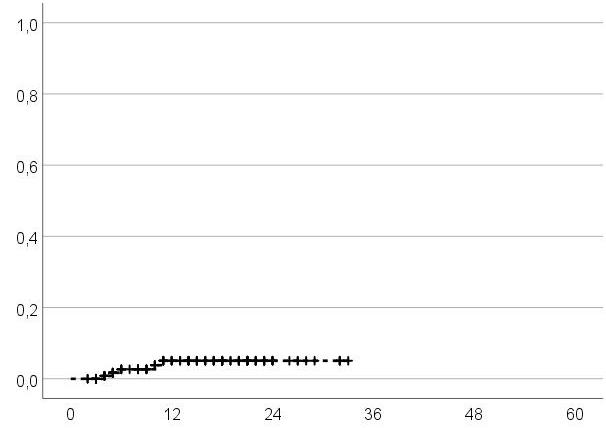


E ***overall GvHD*** F ***significant GvHD***


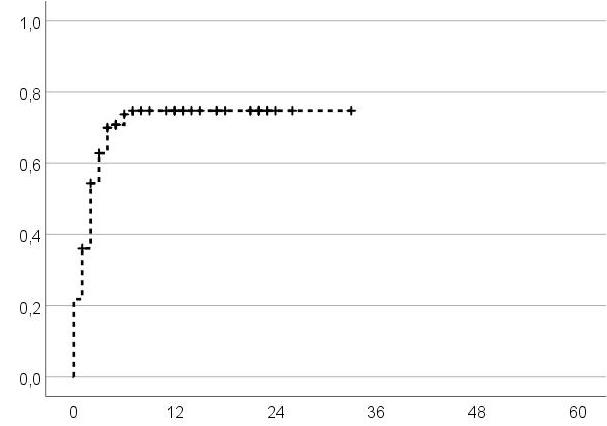

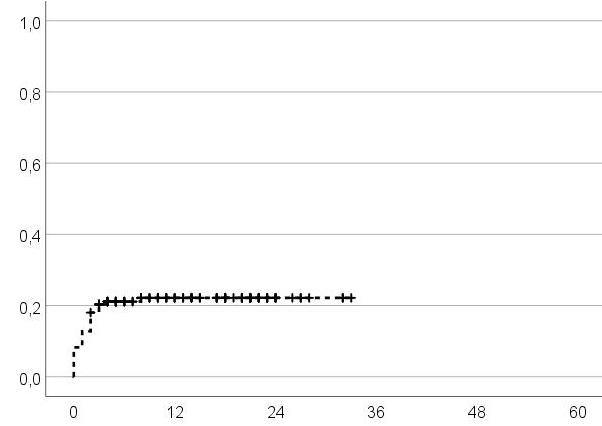


______________________________________________________________________

Legend to Suppl. Figure 1:

Overall Survival (A), Disease-Free Survival (B), Relapse Incidence (C), Non-Relapse Mortality (D), overall GvHD (E), and significant GvHD (F), in hs-chimerism patients without DLI

x-axes: months after alloSCT

**Suppl. Figure 2**

Landmark OS from first DLI with controls by half-decade

______________________________________________________________________

hs-chimerism navigated DLI historical control cohort

A ***OS*** B ***OS***


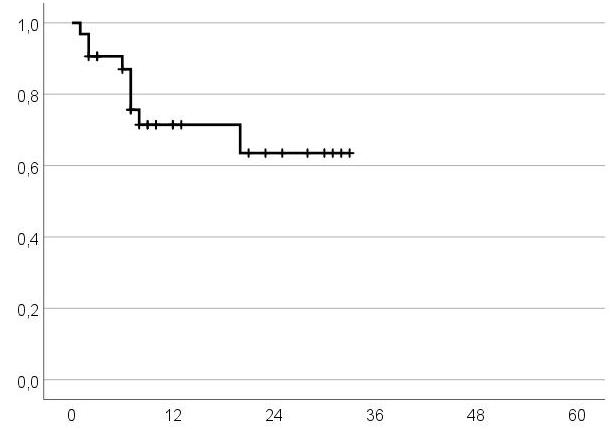

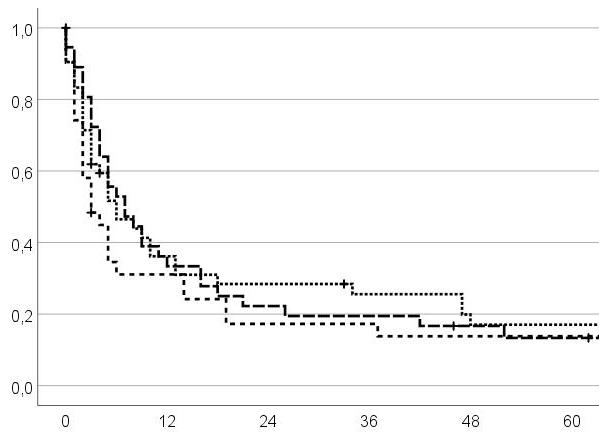


_____ 2019 – 2021 : 2-year-OS 64% ……… 2001 – 2005 : 2-year-OS 28%

- - - - - 2006 – 2010 : 2-year-OS 17%

– – – – 2011 – 2015 : 2-year-OS 22%

Legend to Suppl. Figure 2:

Landmark Overall Survival from first DLI, with controls by half-decade (2001 – 2005 / 2006 – 2010 / 2011 – 2015), in the hs-chimerism navigated group (A) or the historical control group (B)

x-axes: months after first DLI

**Contribution to the field**

Allogeneic stem cell transplantation can cure acute leukemias and other hematological malignancies, mainly through eradication of malignant cells by donor immune cells, called graft-versus-malignancy effect or alloreactivity: a slow, but sustained mechanism. However, if reacting also against normal cells and tissues of the patient, it may lead to potentially devastating graft-versus-host disease. Unfortunately, our capacity to control alloreactivity is limited: it can be reduced with immunosuppressant drugs or by depleting grafts of immune cells, and augmented by withdrawing immunosuppression or by adding donor cells (donor lymphocyte infusions), but remains essentially unpredictable. When leukemias relapse after allogeneic transplantation, donor lymphocytes used to augment alloreactivity are often outpaced by high relapse dynamics. Conversely, even when successfully clearing leukemia, they may induce fatal graft-versus-host disease. In our manuscript, we report on a highly sensitive method to measure patient/donor balance (chimerism), which allows both early relapse prediction and close monitoring (“navigating”) of donor lymphocyte infusions. Our report is the first to show how this method contributed to reduction of further relapses or fatal complications, resulting in substantially improved survival for patients with myeloid malignancies relapsing after allogeneic transplantation. Thus. it represents an advance of precision medicine in the traditionally “imprecise” field of allogeneic transplantation.
